# Aggression in mental health settings: a case study in Ghana

**DOI:** 10.2471/BLT.14.145813

**Published:** 2015-06-05

**Authors:** Helen Jack, Maureen Canavan, Elizabeth Bradley, Angela Ofori-Atta

**Affiliations:** a Institute of Psychiatry, King’s College London, De Crespigny Park, Denmark Hill, London SE5 8AF, England.; b Yale School of Public Health, New Haven, United States of America.; c University of Ghana Medical School, Accra, Ghana.

Stigma towards people living with mental health problems is often the result of deep-rooted fears about irrational behaviour or loss of control.^1^ When we conducted a study of recruitment and retention factors for staff in Ghanaian psychiatric hospitals, we found that the stigma was directed towards mental health professionals too. Some of our respondents linked the pervasive stigma of mental health to perceptions that patients with mental disorders could be aggressive or violent. We used a semi-structured discussion guide with follow-up prompts in face-to-face interviews with 28 mental health workers of all levels. Inclusion criteria were employment in one of Ghana’s three psychiatric hospitals and ability to speak English. We selected respondents using the chain referral method of sampling^2^ to theoretical saturation,^3^ seeking diversity in roles within the hospital, gender, age and length of time working in mental health services.

We asked respondents about daily job activities and reasons why they started and stayed working at a psychiatric hospital. For data analysis, we used the qualitative constant comparative method,^4^ adapted for health services research.^5^ We have described the findings relevant to worker recruitment and retention elsewhere.^6^ Although we asked no questions specifically about stigma or the behaviour of people with mental health problems, many respondents revealed that one of the greatest challenges they encountered at work was fear of and injury from aggression.

Aggressive behaviours of people attending psychiatric hospitals^7^ and primary care settings^8^ have previously been documented. Our work on Ghana’s mental health system suggests that aggression and stigma are central challenges for mental health workers. Appropriate support for mental health professionals could play a key role in reducing stigma, increasing health worker recruitment and retention and improving mental health care. These efforts must be central, not secondary, in global efforts to scale-up access to mental health care.

In low- and middle-income countries, less than 25% of people with the most severe mental disorders receive any conventional treatment. Treatment rates are far lower for people with moderate or mild disorders.^9^ Mental health care, perhaps even more than other areas of medical treatment, relies on trained workers. If the mental health treatment gap is to be addressed, a global effort to strengthen human resources for mental health will be required. There are currently fewer than 1.7 mental health workers of any kind per 100 000 population in in Africa and 5.3 per 100 000 in south-east Asia, compared with 14.8 in the Americas and 43.9 in Europe.^10^ Less than 3% of the medical curriculum is devoted to mental health, leaving many health workers under-prepared to manage people living with mental health problems.^10^ The number of specialized workers, including psychiatrists, psychiatric nurses, counsellors and social workers needs to increase; and other health professionals need to be more involved in managing mental health disorders.^11^

Many participants in our study felt unprepared to manage aggressive behaviour and consequently resorted to inappropriate restraint, overuse of medication or ignoring people with mental health problems. Calling attention to aggressive behaviour could be viewed as blaming the person with mental health problems, adding to stigma. Failing to address the issue, however, threatens both human rights and worker morale. Given the increasing global focus on task-shifting mental health service provision away from specialist workers, all levels of staff who work with patients with mental health disorders should be prepared to manage aggressive behaviour.

The management of aggressive behaviour is not covered by international guidelines on training for, and support of, mental health workers and is largely absent from research on global mental health. Thus far, mental health workforce development materials – including the *WHO Human resources and training in mental health* and the *mhGap intervention guide* *for mental, neurological and substance use disorders in non-specialized health settings* – have insufficient training on managing aggression.^12^^,^^13^ Explicit training on effective and safe handling of aggressive behaviour is most important for frontline workers, such as nurses, nurse assistants and community health workers, who have the most direct contact with people living with mental health disorders. Health professionals in primary care also see people with psychosis, dementia, drug and alcohol dependence and other conditions that can lead to aggression. Physicians, hospital managers and others in supervisory roles should also receive training on how to lead organizational responses to aggression, which target the causes of aggressive behaviour, such as overcrowding or inappropriate medication use, and help build a safe work environment.^14^

Clinical management protocols could be useful in management of aggressive behaviour. These types of protocols have been helpful in the context of human immunodeficiency virus (HIV) care, where a protocol for emergency prophylaxis is increasingly standard practice. Even if protocols are infrequently used, having them in place can ease health worker anxiety and as a result, reduce stigma towards service users.^15^^,^^16^ The field of global mental health could also borrow other strategies from work on HIV stigma, which includes mass media campaigns featuring testimonials of people living with HIV, structural interventions in hospitals or other organizations to ensure that people living with HIV are fully integrated; large scale-up of access to treatment and development and evaluation of many innovative education interventions for health workers and communities.^17^

While the call for an expanded mental health workforce is well founded, we must also consider workforce development efforts to address the unique and sensitive challenges that mental health workers tackle on a daily basis. Mechanisms for helping mental health workers handle stigma, job stress and aggression are paramount, particularly as efforts to integrate mental health into primary care and increase the involvement of non-specialist workers are scaled up.^11^ Issues of stigmatization and aggression must be addressed with careful planning and discussion among community representatives, educators, clinicians and researchers.
